# Association of low body mass index with the risk of major adverse cardiovascular events among patients with acute myocardial infarction: a single-center cohort study from Iran

**DOI:** 10.1186/s12872-026-05739-3

**Published:** 2026-03-29

**Authors:** Abbas Andishmand, Marzieh Azimizadeh, Seyedeh Mahdieh Namayandeh, Hamidreza Mohammadi, Mohammad Hossein Mirjalili

**Affiliations:** 1 Yazd Cardiovascular Research Center, Non-communicable Diseases Research Institute, Shahid Sadoughi University of Medical Sciences, Afshar Hospital, Jomhouri Blvd., Yazd, Iran; 2 Department of Epidemiology, Shahid Sadoughi University of Medical Sciences, Yazd, Iran

**Keywords:** Body Mass Index, Acute Myocardial Infarction, Major Adverse Cardiovascular Events

## Abstract

**Background:**

The association between body mass index (BMI) and outcomes after acute myocardial infarction (AMI) remains controversial. While obesity is a known cardiovascular risk factor, low BMI may also have prognostic implications. This study aimed to investigate the association between BMI categories and major adverse cardiovascular events (MACE) in AMI patients.

**Methods:**

This retrospective cohort study included 1,018 AMI patients admitted to Afshar Hospital, Yazd, Iran. Patients were categorized into four BMI groups: underweight (< 18.5 kg/m²), normal weight, overweight, and obese. Demographic information along with risk factors, clinical history, and clinical presentation, were extracted. MACE was defined as all-cause mortality, AMI, ischemic stroke, cardiovascular hospitalizations, and revascularization procedures. Univariate and multivariate logistic regression analyses and Restricted cubic spline (RCS) regression method with four knots were used to assess the relationships between BMI and outcomes.

**Results:**

Underweight patients (11.3%) had significantly higher mortality (24.3%) compared to other BMI groups (*P* < 0.001). After adjustment, underweight remained an independent risk factor for mortality (OR 3.12, 95% CI 1.42–6.89, *p* = 0.005), but not for MACE. No significant associations were found between overweight or obesity and increased risk of MACE or mortality. The dose-response analysis revealed a U-shaped curve for mortality (P for nonlinearity < 0.001), but not for MACE (*P* = 0.12).

**Conclusion:**

While obesity is a well-established cardiovascular risk factor, our findings suggest that low BMI is associated with increased one-year mortality in patients with AMI, but not with MACE after adjustment. These results should be interpreted with caution, as low BMI may reflect underlying health status rather than a direct causal factor. Further studies are needed to clarify the mechanisms underlying this association.

## Introduction

Acute myocardial infarction (AMI) is a common clinical acute and severe disease with rapid onset, rapid progression, and high mortality [1, 2]. It is one of the leading causes of mortality worldwide, and its prevalence continues to rise, particularly in low- and middle-income countries, where rapid urbanization and lifestyle changes have led to a higher burden of cardiovascular disease (CVD) [3, 4]. The advancement of medical technologies, including percutaneous coronary intervention (PCI), has significantly reduced the immediate mortality rates of AMI. However, the incidence of major adverse cardiovascular events (MACE) among patients after PCI remains high [5, 6].

Following an AMI, patients face an elevated risk of recurrent cardiovascular events, including heart failure, stroke, and sudden cardiac death. These risks are influenced by several factors, including the quality of care, adherence to post-AMI treatment, lifestyle changes, and the presence of underlying risk factors such as hypertension (HTN), diabetes mellitus (DM), and hyperlipidemia (HLP) [7, 8]. Among these factors, the association between body mass index (BMI) and cardiovascular outcomes is complex and not fully understood. While several studies have suggested a U-shaped relationship between BMI and cardiovascular risk, with both low and high BMI associated with poor outcomes [9, 10].

Traditionally, a high BMI has been regarded as a well-established risk factor for the development of coronary artery disease (CAD) and other cardiovascular conditions [11, 12]. However, recent studies have drawn attention to the potential risks associated with low BMI. Recent studies have shown, Low BMI, which may signal malnutrition or frailty, has emerged as a possible independent risk factor for poor clinical outcomes following AMI, including increased mortality and a higher likelihood of recurrent cardiovascular events [13–15].

In contrast, studies, suggest that a higher BMI may provide some protective advantage in the long-term outcomes of AMI patients [16]. Soyoon Park et al. reported that higher BMI levels in Asian populations undergoing PCI were associated with better long-term outcomes [17].

Patients with low BMI are often older, frailer, have worse left ventricular ejection fraction (LVEF), and have more comorbidities, suggesting sarcopenia, cachexia, or chronic disease rather than healthy leanness [18–20]. Furthermore, BMI is not simply a marker of body fat, but is also associated with various biological and metabolic changes that affect cardiovascular health, including dyslipidemia, insulin resistance, and inflammation [21, 22].

As the global prevalence of cardiovascular disease continues to rise, especially in developing countries, identifying modifiable risk factors such as BMI and improving patient outcomes after PCI remain crucial goals in cardiovascular care [23–25]. Previous studies have been conducted in high- and upper-middle-income countries in Europe, North America, and East Asia, but there have been no significant studies in West Asian populations where lifestyle, diet, and access to healthcare differ significantly from other regions. We aim to determine whether low BMI is an independent risk factor for poor clinical outcomes, including MACE, and how it compares to the effects of high BMI.

## Methods

### Study design and populations

This retrospective cohort study was conducted to investigate the associations between baseline body weight, clinical history, clinical presentation, and MACE at one-year follow-up. This study enrolled AMI patients at Afshar Hospital affiliated to Shahid Sadoughi University of Medical Sciences (Yazd, Iran) from January 2018 to September 2022. The patients who were at least 18 years of age were included. Patients with incomplete medical records, an unknown type of infarction, acute or chronic infection or inflammation, malignant tumors, autoimmune disease, hematologic disease were excluded were excluded from the study. Out of the total of 1264 patients discharged from the hospital with a diagnosis of AMI during the specified period, 1018 patients were included in this analysis. All patients were followed up for 12 months after discharge, either at Afshar Hospital’s preventive heart clinic or by telephone to check their medical status and record the date of death.

### Ethical approval

This study adhered to the principles outlined in the Declaration of Helsinki and received approval from the local ethics committee of Shahid Sadoughi University of Medical Sciences (IR.SSU.MEDICINE.REC.1401.110). The data collection process ensured complete anonymity, with no personally identifying information included in the individual data.

### Data collection

Data were obtained from the electronic medical records and data bank of Afshar Hospital, as well as the MI Registry Bank of the Cardiovascular Research Center of Afshar Hospital. The collected variables included age, sex, height, weight, risk factors for CAD such as hypertension (HTN), diabetes mellitus (DM), hyperlipidemia (HLP), smoking, addiction, family history of CAD, cerebrovascular accident (CVA), history of PCI or coronary artery bypass grafting (CABG), type of MI (STEMI or NSTEMI), serum creatinine level, and LVEF. The diagnosis of AMI was based on the third universal definition of MI [26]. BMI is calculated as the ratio of body weight to height squared. BMI according to the WHO definition was classified into four categories: underweight (<18.5 kg/m²), normal weight (18.5–24.9 kg/m²), overweight (25–29.9 kg/m²), and obesity (≥30 kg/m²). However, due to the low prevalence of underweight individuals in our study, and consistent with other studies, we categorized BMI <22.0 kg/m² as low BMI, and a BMI of 22.0–24.9 kg/m² as the reference group for normal weight [27, 28]. The estimated glomerular filtration rate (eGFR) was calculated using the Modification of Diet in Renal Disease (MDRD) formula [29].

### Outcomes

The outcomes were one-year MACE. MACE was defined as the composite of all-cause of mortality, AMI, ischemic stroke, Cardiovascular hospitalizations, and Revascularization, including PCI, and emergency CABG. Cardiovascular hospitalization was defined as any hospitalization where the primary cause was related to cardiovascular events, including AMI, heart failure, or stroke. All-cause mortality was considered in this study, meaning that any death, regardless of cause, was included in the analysis. The assessment of MACE was performed through a detailed chart review of provider notes at the time of the event. In cases where additional clarification was needed, adjudication was conducted by one of the co-authors specializing in cardiovascular outcomes.

### Statistical analysis

Statistical analysis was performed using SPSS version 26 (IBM Inc., NJ, USA) and R version 4.2.2 (www.R-project.org). Continuous variables were expressed as means and standard deviations, while categorical variables were presented as frequencies and percentages. One-way ANOVA was used for quantitative data, while chi-square and Fisher's exact tests were employed for qualitative data. Univariate and multivariate binary logistic regression were performed. The association between BMI and AMI outcomes was assessed using multivariate logistic regression models, adjusting for potential confounders. Initially, age and sex were modeled, followed by a full-adjustment model to account for all influencing factors. Subgroup analyses were conducted to explore the impact of individual patient characteristics on the relationship between BMI and MI outcomes. In addition, Restricted cubic spline (RCS) regression method with four knots were used to assess the dose-response relationships between BMI as a continuous variable and outcomes. The findings of these analyses were reported in terms of odds ratios (OR), along with corresponding 95% confidence intervals (95% CI). A two-tailed *P*-value below 0.05 was deemed statistically significant.

## Results

A total of 1,018 patients were enrolled in the study mean age was 74.8 years and 62.1 % male. In all, 115 patients (11.3%) were classified as underweight (BMI <22.0 kg/m²), 302 (29.7%) as normal weight (22.0–24.9 kg/m²), 417 (41.0%) as overweight, and 184 (18.1%) as obese. The baseline characteristics of the study population were analyzed in different BMI categories. Age, sex distribution, risk factors such as DM, HTN, HLP, smoking, addiction, prior cardiovascular interventions including PCI, and CABG, CVA history, clinical presentation (NSTEMI vs STEMI), LVEF, and renal function did not show significant differences across BMI groups (all *P*>0.05). However, Patients with underweight were more likely to report a history of PCI than all other BMI categories (*P* <0.05). The distribution of age ≥65 years also differed significantly across BMI groups (*P*-value = 0.025), with the highest frequency observed in the BMI <22 group. (Table 1).

**Table 1:** Baseline characteristics and clinical presentation

Variable	Underweight BMI < 22 (*N* = 115)	Normal 22-24.9 (*N* = 302)	Overweight 25-29.9 (*N* = 417)	Obesity ≥ 30 (*N* = 184)	Total (*N* = 1018)	*P*-Value
General characteristics						
Age (years)	64.77 ± 12.2	62.35 ± 13.2	61.8 ± 12.3	62 ± 12.3	62.3 ± 12.6	0.16
Age ≥ 65	65 (56.5)	141 (46.7)	174 (41.7)	76 (41.3)	456 (44.8)	0.025
Sex, Male	81 (70.4)	211 (69.9)	280 (67.1)	129 (70.1)	701 (68.9)	0.8
BMI (Kg/m^2^)	19.6 ± 1.48	23.6 ± 0.77	27.32 ± 1.3	33.2 ± 2.7	26.4 ± 4.33	< 0.001
Risk factors						
DM	40 (34.8)	90 (29.8)	132 (31.7)	63 (34.2)	325 (31.9)	0.67
HTN	57 (49.6)	126 (41.7)	183 (43.9)	87 (47.3)	453 (44.5)	0.42
HLP	52 (45.2)	110 (36.4)	168 (40.3)	80 (43.5)	410 (40.3)	0.28
Smoking	34 (29.6)	93 (30.8)	130 (31.2)	66 (35.9)	323 (31.7)	0.59
Addiction	22 (19.1)	49 (16.2)	73 (17.5)	38 (20.7)	182 (17.9)	0.63
Family History of CAD	35 (30.4)	80 (26.5)	136 (32.6)	60 (32.6)	311 (30.6)	0.31
Clinical history						
Prior PCI	24 (20.9)	25 (8.3)	60 (14.4)	25 (13.6)	134 (13.2)	0.005
Prior CABG	10 (8.7)	19 (6.3)	29 (7.0)	10 (5.4)	68 (6.7)	0.72
Prior CVA	5 (4.3)	7 (2.3)	14 (3.4)	3 (1.6)	29 (2.8)	0.45
Clinical presentation						
NSTEMI	48 (41.7)	124 (41.1)	167 (40.0)	73 (39.7)	412 (40.5)	0.97
STEMI	67 (58.3)	178 (58.9)	250 (60.0)	111 (60.3)	606 (59.5)	
LVEF (%)	42 ± 10.64	41.25 ± 11	42 ± 9.85	39.9 ± 9.8	41.4 ± 10.3	0.38
LVEF < 40	29 (25.2)	78 (25.8)	95 (22.8)	54 (29.3)	256 (25.1)	0.33
eGFR (mL/min/1.73m2)	60.9 ± 16.96	61.75 ± 17.74	59.7 ± 16.9	62 ± 15.8	60.8 ± 16.9	0.52
eGFR < 60	36 (31.3)	82 (27.2)	142 (34.1)	63 (34.2)	323 (31.7)	0.21

Data presented as mean ± standard deviation or number (%)

*Abbreviations*: *BMI* body mass index, *CAD* coronary artery disease, *DM* diabetes mellitus, *HTN* hypertension, *HLP* hyperlipidemia, *PCI* percutaneous coronary intervention, *CABG* coronary artery bypass grafting, *CVA* cerebrovascular accident, *STEMI* ST-elevation myocardial infarction, *NSTEMI* non-ST elevation myocardial infarction, *LVEF* left ventricular ejection fraction, *eGFR* estimated glomerular filtration rate

As shown in Table 2, the incidence of MACE was highest in patients with underweight (33%), followed by those with obesity (32.1%), although this difference was not statistically significant (*P* = 0.11). Mortality (24.3%) was significantly more common in the underweight group compared to all other BMI categories (*P* < 0.001). (Table 2).

**Table 2:** major adverse cardiovascular events (MACE) among patients according to BMI

	Underweight BMI < 22 (*N* = 115)	Normal 22-24.9 (*N* = 302)	Overweight 25-29.9 (*N* = 417)	Obesity ≥ 30 (*N* = 184)	Total (*N* = 1018)	*P*-Value
MACE	38 (33.0)	70 (23.2)	107 (25.7)	59 (32.1)	274 (26.9)	0.067
mortality	18 (15.7)	15 (5.0)	22 (5.3)	17 (9.2)	72 (7.1)	0.001
Acute MI	4 (3.5)	3 (1.0)	3 (0.7)	1 (0.5)	11 (1.1)	0.16
Ischemic stroke	1 (0.9)	2 (0.7)	2 (0.5)	2 (1.1)	7 (0.7)	0.86
Cardiovascular hospitalizations	15 (13.0)	50 (16.6)	83 (19.9)	40 (21.7)	188 (18.5)	0.18
Revascularization^†^	12 (10.4)	34 (11.3)	42 (10.1)	18 (9.8)	106 (10.4)	0.94

*BMI* body mass index, *MACE* major adverse cardiovascular event, *MI* myocardial infarction

† Include coronary stenting and bypass surgery

**Fig. 1:**
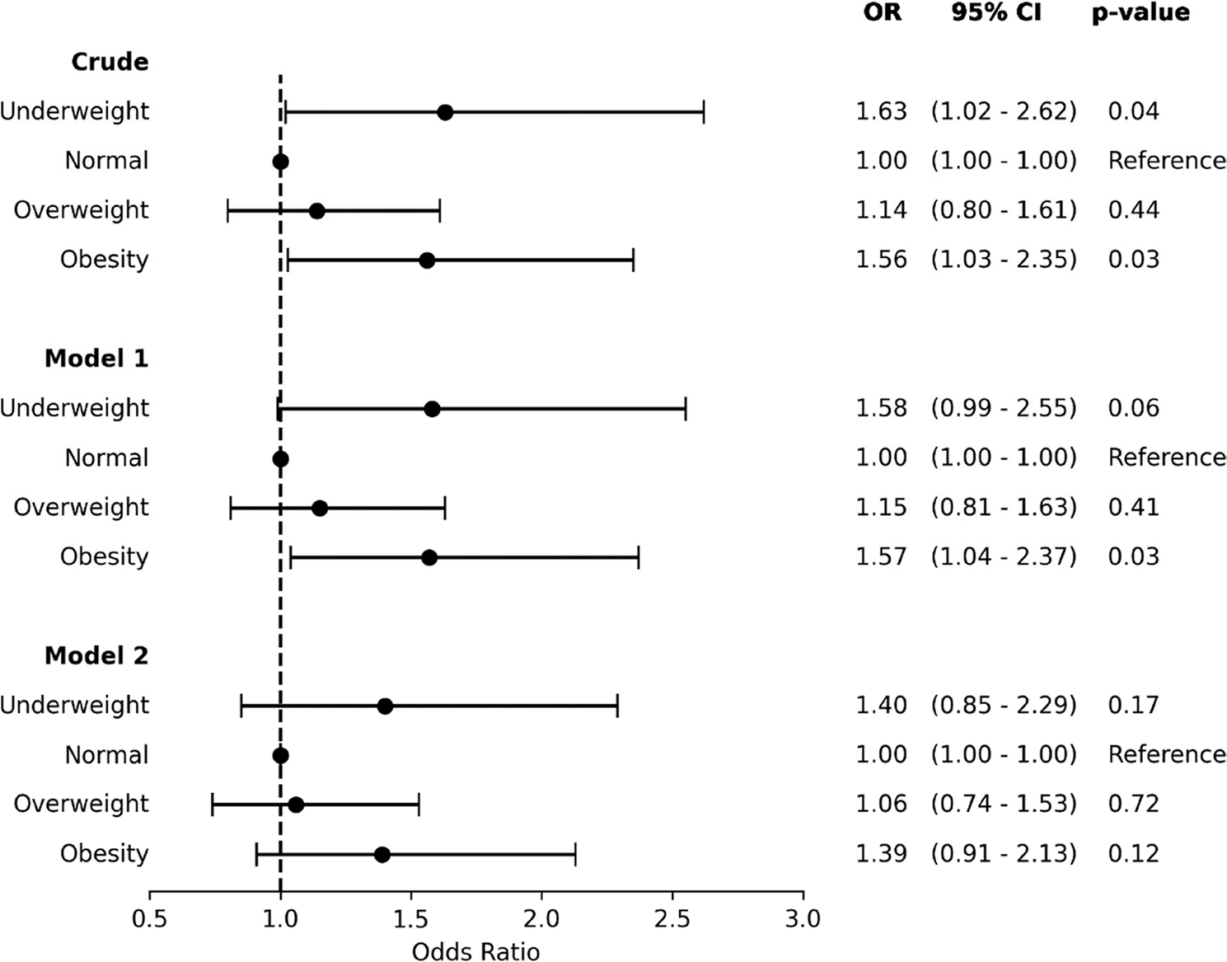
Risk of MACE according to BMI Categories. The results are expressed as Odds Ratios and 95% confidence intervals. Model I: adjusted for age and sex. Model II: adjusted for variables included in model I and risk factors, clinical history, clinical presentation, LVEF, and eGFR. OR = Odds Ratio; 95% CI = 95% confidence interval

In the crude model, underweight individuals had a significantly higher risk of MACE (OR 1.63, , 95% CI 1.02 – 2.62, p = 0.04), but this association became non-significant after adjusting for age and sex (Model I) and further adjusting all variables (Model II). In individuals with obesity, the risk of MACE was significant in both the crude model and Model I (OR 1.57, 95% CI 1.04 - 2.37, *p* = 0.03), but it became non-significant after further adjustment for clinical factors (OR 1.39, 95% CI 0.91 - 2.13, *p* = 0.12). (Fig. 1).

**Fig. 2:**
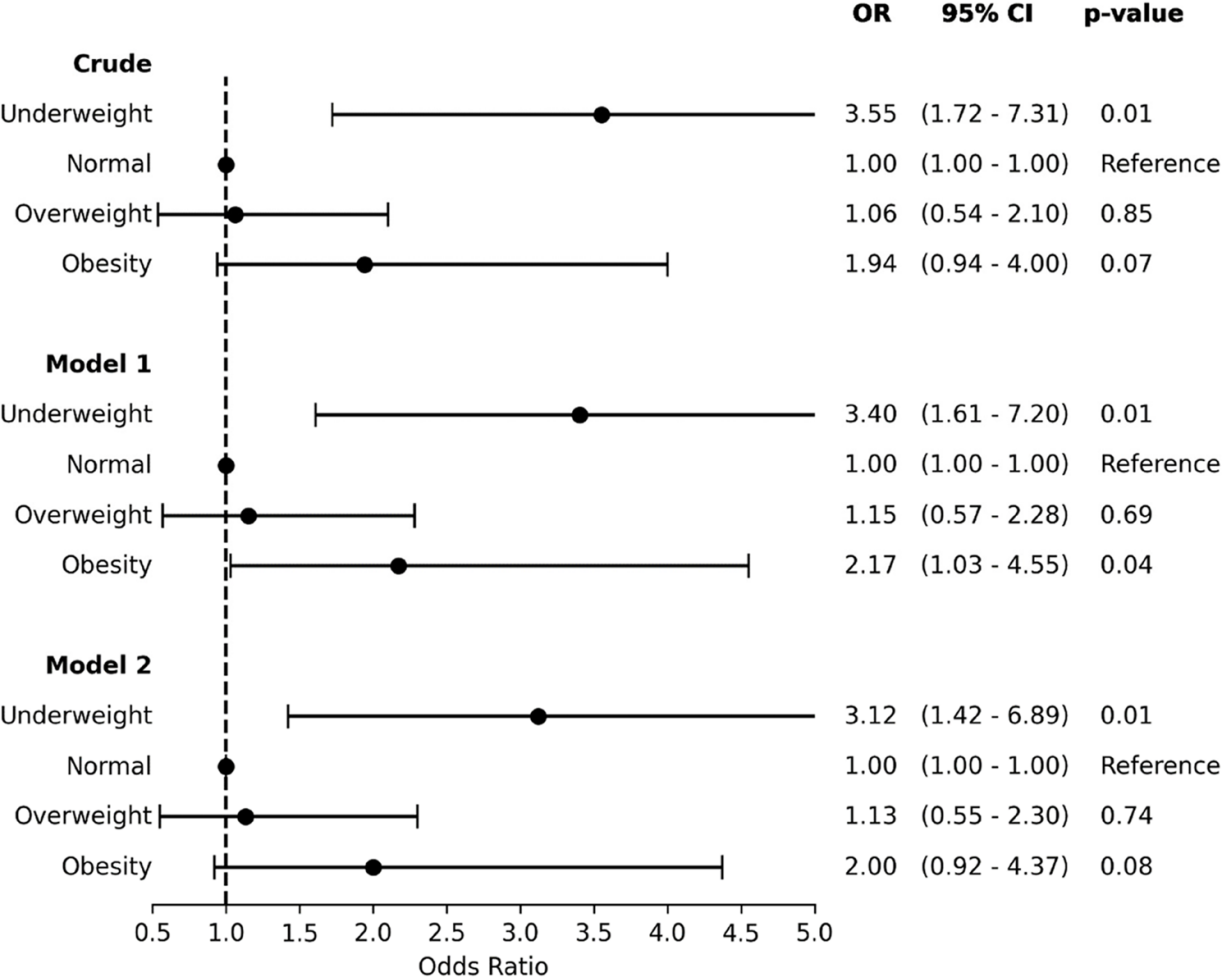
Risk of Mortality according to BMI Categories. The results are expressed as Odds Ratios and 95% confidence intervals. Model I: adjusted for age and sex. Model II: adjusted for variables included in model I and risk factors, clinical history, clinical presentation, LVEF, and eGFR. OR = Odds Ratio; 95% CI = 95% confidence interval

For individuals with underweight, the crude model showed a significantly higher risk of mortality (OR 3.55, 95% CI 1.72–7.31, *p* < 0.001). After full adjustment, the risk remained significant (OR 3.12, 95% CI 1.42–6.89, *p* = 0.005). In contrast, obesity was not significantly associated with mortality in either the crude or fully adjusted model (OR 2.00, 95% CI 0.92–4.37, *p* = 0.08). (Fig. 2).

**Fig. 3:**
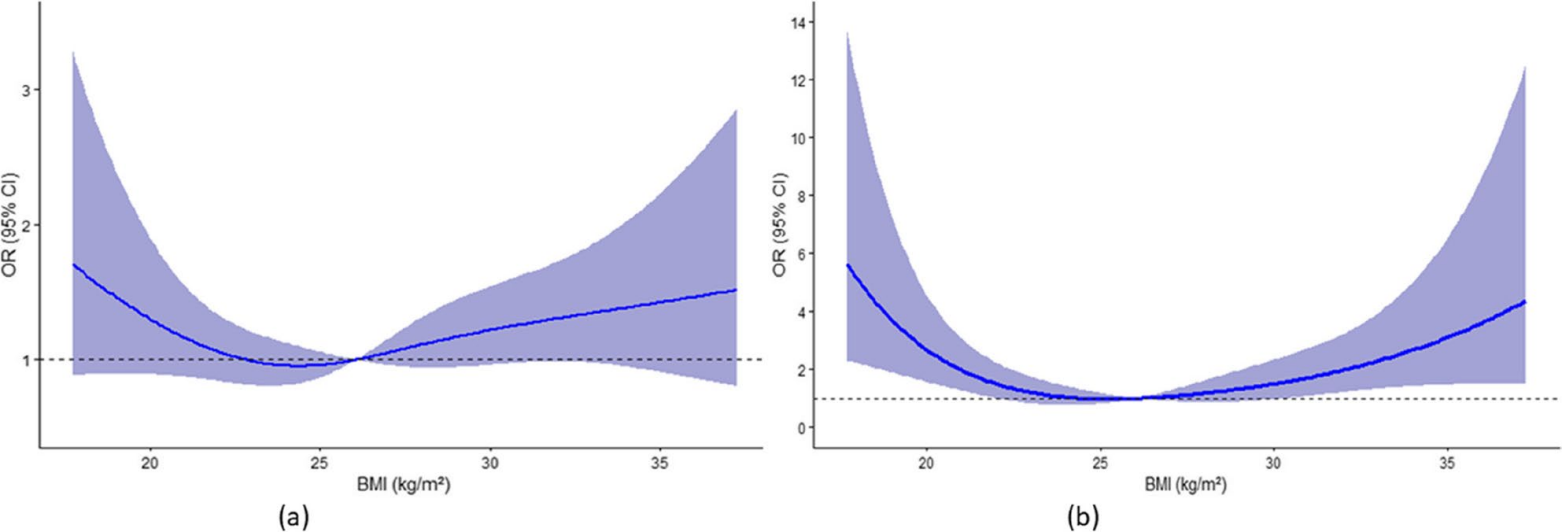
Dose-response relationship between BMI and MACE (**a**) and all-cause mortality (**b**), in the fully adjusted model

The dose-response analysis revealed a U-shaped curve between BMI and the risk of MACE and all-cause mortality after full adjusting. The nonlinear trend was significant for mortality (*P* for nonlinearity < 0.001), but not for MACE (*P* for nonlinearity = 0.12). (Fig. 3).

## Discussion

Our study demonstrated that underweight status was strongly associated with increased one-year mortality after AMI. In contrast, overweight and obesity were not associated with mortality and did not provide a survival advantage compared to normal BMI. Thus, our findings do not provide direct evidence of an “obesity paradox”; instead, they underscore the markedly adverse prognosis associated with low BMI. These findings are consistent with prior studies reporting that, although higher BMI is a recognized risk factor for CVD, higher BMI may not always be an independent predictor of post-AMI prognosis [30–33]. For instance, Kaneko et al. demonstrated that patients with overweight or obesity had a lower risk of all-cause mortality and MACE after PCI [34]. Firman et al. observed patients with obesity showing a reduced incidence of MACE [35]. Similarly, large meta-analyses have confirmed that patients with overweight and obesity who have CAD or are undergoing PCI often experience lower short- and long-term mortality compared to those with normal BMI [36–38]. The persistence of this findings, as reported by Lin et al., reinforces the possibility of underlying protective mechanisms in higher BMI categories [27]. However, more recent studies had highlighted that obesity paradox may be driven by the poor outcomes of underweight or frail patients rather than by a true protective effect of obesity [39]. In line with this interpretation, our findings primarily point to the vulnerability of patients with low BMI.

It is important to acknowledge that the association between BMI and mortality is intricate and subject to variation based on the characteristics of the population and the specific medical condition under scrutiny [40]. In our cohort, patients with overweight and obesity did not exhibit increased mortality compared to individuals with normal weight, whereas underweight status likely reflecting frailty, chronic disease, or illness-related weight loss was associated with worse outcomes. Because we did not collect detailed data on nutritional status, body composition, functional capacity, or treatment intensity, we cannot infer causality or determine which specific mechanisms are responsible for these associations. Several mechanisms could explain the adverse prognosis in patients with underweight. As highlighted by recent studies, Low BMI may be associated with conditions such as malnutrition, sarcopenia, or chronic inflammation, which could potentially contribute to worse outcomes; however, these factors were not directly assessed in the present study [41, 42]. Sarcopenia, characterized by the loss of muscle mass and strength, can lead to decreased mobility and impaired cardiac function, which are both associated with poor clinical outcomes following AMI [41]. Moreover, chronic inflammation, a common feature in elderly patients with underweight and comorbidities, is linked to an increased risk of cardiovascular events [43, 44].

In our study, elderly patients had a higher frequency in the underweight group. Elderly patients with underweight had significantly higher risks of mortality, consistent with previous reports that low BMI in this group often reflects sarcopenia, cachexia, malnutrition, malignancy, or other chronic illnesses [45, 46].

In addition, the nutritional deficiency often seen in underweight individuals can impair immune function and increase susceptibility to infections, which can worsen outcomes post-AMI [47, 48]. These factors suggest that underweight may act as a surrogate marker for frailty or malnutrition rather than simply indicating a healthy low body weight [49].

In the present study, we also observed that despite the higher frequency of low BMI among elderly patients, these individuals had more comorbidities. These conditions may contribute to vulnerability to both cardiovascular and non-cardiovascular deaths, but we did not have sufficiently granular information on medication doses, adherence, or rehabilitation to examine differences in treatment intensity across BMI categories. Any suggestion that patients with overweight and obesity received more aggressive care in our cohort therefore remains speculative [50].

One of the most intriguing findings in our study was the observation that underweight status predicted mortality but did not predict MACE after adjusting for confounders. This discrepancy may be attributed to the fact that mortality is a more direct and definitive endpoint than MACE, which includes a broader range of cardiovascular events such as stroke, reinfarction, and rehospitalization. As suggested by recent literature, the poor prognosis in underweight individuals may reflect the combined effect of frailty, malnutrition, and inflammatory state, which contributes to a higher risk of death but does not necessarily translate into a higher incidence of MACE. Furthermore, other variables, such as renal function, LVEF, and the presence of comorbidities, likely play a more significant role in the development of MACE. Our study showed no significant difference in these factors across BMI categories, except for the underweight group, which had a higher prevalence of prior PCI and other comorbid conditions. These findings underscore the complexity of cardiovascular risk and suggest that BMI alone is insufficient to capture the full spectrum of risk factors contributing to MACE.

Sex differences are an important consideration in understanding the outcomes of cardiovascular diseases, including AMI. While previous research has shown that malnutrition and BMI can affect prognosis differently in men and women, our study did not find significant sex-based differences in the relationship between nutritional status and in-hospital mortality. However, it is known that women may have different body compositions and hormonal profiles compared to men, which could influence how nutritional status impacts their cardiovascular outcomes. Although this study did not specifically address gender differences in depth, further research with larger sample sizes is needed to explore how these factors interact with nutritional status to influence mortality in ACS patients [47, 51, 52].

Importantly, BMI has significant limitations as a measure of adiposity. It does not differentiate between fat mass and fat-free mass, and misclassification may occur [36, 53, 54]. For example, a lower BMI could be related to sarcopenia rather than low fat mass, while being overweight may reflect increased muscle mass, rather than excessive adipose tissue. Patients with sarcopenia tend to have limited exercise capacity and reduced mobility, which are both associated with increased total mortality [55].

The findings of this study have important clinical implications, suggesting that the relationship between BMI and mortality in the context of AMI is complex and may differ from the general understanding of the impact of excess weight on health outcomes. our key finding is the markedly worse prognosis of patients with underweight. Clinicians should therefore be particularly attentive to underweight AMI patients, in whom low BMI may be a marker of frailty or occult disease. Alternative measures, such as waist circumference or body fat percentage, may provide more accurate risk stratification. Further research is warranted to clarify the mechanisms linking both low and high BMI with cardiovascular outcomes after AMI and to determine to what extent an apparent “obesity paradox” reflects confounding and selection biases rather than true causality [56].

## Limitations

This study has several limitations. First, its retrospective design may introduce selection and information bias. Second, the analysis was limited to a single tertiary care center, which may affect the generalizability of the findings to other populations. Third, the relationship between BMI and cardiovascular outcomes can be influenced by unmeasured confounding factors such as nutritional status, inflammatory markers, and socioeconomic variables, which were not accounted for in this study. In addition, other indicators should be considered, such as SGA, MNA, or body composition measurements, to provide a more comprehensive assessment of nutritional status and body composition. Since nutritional status, sarcopenia, and inflammatory markers were not directly measured, no causal inferences can be made regarding the mechanisms underlying the observed association. Finally, BMI as a single metric does not fully capture body composition or fat distribution, which may limit the accuracy of obesity-related risk assessment.

Moreover, the small sample size in the underweight group and the unclear cause of mortality further limits the interpretation of the results. Prospective studies exploring the influence of confounding factors and investigating the role of different adipose tissue distributions and metabolic factors in the context of AMI would provide a more comprehensive understanding of this phenomenon and help clarify whether an apparent obesity paradox reflects true biological protection or the adverse impact of low BMI.

## Conclusion

This study demonstrated that underweight was significantly associated with one-year mortality in patients with AMI. In contrast, no independent association was observed between BMI and MACE after adjustment. Overweight and obesity were not associated with increased mortality compared with normal BMI, and no evidence of an obesity paradox was observed.

These findings should be interpreted with caution, as low BMI may reflect unmeasured factors such as frailty, comorbid conditions, or overall health status, rather than a direct causal risk factor for mortality, and may also be influenced by reverse causation. Therefore, BMI may be considered a marker of vulnerability rather than an independent determinant of adverse outcomes. Further prospective studies incorporating detailed assessments of body composition, frailty, and nutritional status are needed to clarify the underlying mechanisms.

## Data Availability

The datasets used and/or analysed during the current study are available from the corresponding author on reasonable request.
